# Comprehensive validation of early diagnostic algorithms for myocardial infarction in the emergency department

**DOI:** 10.1093/qjmed/hcad242

**Published:** 2023-10-25

**Authors:** M Tada, H Matano, H Azuma, K -I Kano, S Maeda, S Fujino, N Yamada, H Uzui, H Tada, K Maeno, Y Shimada, H Yoshida, M Ando, T Ichihashi, Y Murakami, Y Homma, H Funakoshi, K Obunai, A Matsushima, N Ohte, A Takeuchi, Y Takada, S Matsukubo, H Ando, Y Furukawa, A Kuriyama, T Fujisawa, A R Chapman, N L Mills, H Hayashi, N Watanabe, T A Furukawa

**Affiliations:** Department of Emergency Medicine, Nagoya City University East Medical Center, Aichi, Japan; Department of Neurology, Nagoya City University East Medical Center, Aichi, Japan; Department of Health Promotion and Human Behavior, Kyoto University Graduate School of Medicine/School of Public Health, Kyoto, Japan; Department of Emergency Medicine, Fukui-Ken Saiseikai Hospital, Fukui, Japan; Department of Emergency Medicine, Fukui Prefectural Hospital, Fukui, Japan; Department of Emergency Medicine, Fukui Prefectural Hospital, Fukui, Japan; Department of Emergency Medicine, Fukui Prefectural Hospital, Fukui, Japan; Department of Cardiology, Vascular Center, Fukui Prefectural Hospital, Fukui, Japan; Department of Emergency Medicine, University of Fukui, Fukui, Japan; Department of Cardiovascular Medicine, University of Fukui, Fukui, Japan; Department of Cardiovascular Medicine, University of Fukui, Fukui, Japan; Department of Cardiology, Fukui-Ken Saiseikai Hospital, Fukui, Japan; Department of Emergency Medicine, Japanese Red Cross Fukui Hospital, Fukui, Japan; Department of Cardiology, Japanese Red Cross Fukui Hospital, Fukui, Japan; Department of Emergency and Critical Care Medicine, Kariya Toyota General Hospital, Aichi, Japan; Department of Cardiology, Nagoya City University East Medical Center, Aichi, Japan; Department of Cardiology, Nagoya City University East Medical Center, Aichi, Japan; Department of Emergency Medicine, Chiba Kaihin Municipal Hospital, Chiba, Japan; Department of Emergency and Critical Care Medicine, Tokyo Bay Urayasu Ichikawa Medical Center, Chiba, Japan; Department of Cardiology, Tokyo Bay Urayasu Ichikawa Medical Center, Chiba, Japan; Department of Emergency Medicine and Critical Care, Nagoya City University Graduate School of Medical Sciences, Aichi, Japan; Department of Cardiology, Nagoya City University Graduate School of Medicine, Aichi, Japan; Department of Emergency Medicine, Konan Kosei Hospital, Aichi, Japan; Department of Cardiology, Konan Kosei Hospital, Aichi, Japan; Department of Emergency Medicine and General Internal Medicine, Social Medical Corporation Kyouryoukai Ichinomiya Nishi Hospital, Aichi, Japan; Department of Emergency Medicine and General Internal Medicine, Social Medical Corporation Kyouryoukai Ichinomiya Nishi Hospital, Aichi, Japan; Department of Cardiology, Social Medical Corporation Kyouryoukai Ichinomiya Nishi Hospital, Aichi, Japan; Department of Primary Care and Emergency Medicine, Kyoto University Graduate School of Medicine, Kyoto, Japan; British Heart Foundation Center for Cardiovascular Science, University of Edinburgh, Edinburgh, UK; British Heart Foundation Center for Cardiovascular Science, University of Edinburgh, Edinburgh, UK; British Heart Foundation Center for Cardiovascular Science, University of Edinburgh, Edinburgh, UK; Usher Institute of Population Health Sciences and Informatics, University of Edinburgh, Edinburgh, UK; Department of Emergency Medicine, University of Fukui, Fukui, Japan; Department of Psychiatry, Soseikai General Hospital, Kyoto, Japan; Department of Health Promotion and Human Behavior, Kyoto University Graduate School of Medicine/School of Public Health, Kyoto, Japan

## Abstract

**Objective:**

To comprehensively evaluate diagnostic algorithms for myocardial infarction using a high-sensitivity cardiac troponin I (hs-cTnI) assay.

**Patients and methods:**

We prospectively enrolled patients with suspected myocardial infarction without ST-segment elevation from nine emergency departments in Japan. The diagnostic algorithms evaluated: (i) based on hs-cTnI alone, such as the European Society of Cardiology (ESC) 0/1-h or 0/2-h and High-STEACS pathways; or (ii) used medical history and physical findings, such as the ADAPT, EDACS, HEART, and GRACE pathways. We evaluated the negative predictive value (NPV), sensitivity as safety measures, and proportion of patients classified as low or high-risk as an efficiency measure for a primary outcome of type 1 myocardial infarction or cardiac death within 30 days.

**Results:**

We included 437 patients, and the hs-cTnI was collected at 0 and 1 hours in 407 patients and at 0 and 2 hours in 394. The primary outcome occurred in 8.1% (33/407) and 6.9% (27/394) of patients, respectively. All the algorithms classified low-risk patients without missing those with the primary outcome, except for the GRACE pathway. The hs-cTnI-based algorithms classified more patients as low-risk: the ESC 0/1-h 45.7%; the ESC 0/2-h 50.5%; the High-STEACS pathway 68.5%, than those using history and physical findings (15–30%). The High-STEACS pathway ruled out more patients (20.5%) by hs-cTnI measurement at 0 hours than the ESC 0/1-h and 0/2-h algorithms (7.4%).

**Conclusions:**

The hs-cTnI algorithms, especially the High-STEACS pathway, had excellent safety performance for the early diagnosis of myocardial infarction and offered the greatest improvement in efficiency.

## Introduction

There are many diagnostic algorithms for myocardial infarction in the emergency department. Traditionally, algorithms using medical history and physical findings, such as the HEART (History, Electrocardiography [ECG], Age, Risk factors, Troponin) pathway, have been the standard.^1^ In contrast, increasing attention has been given to algorithms based on high-sensitivity cardiac troponin (hs-cTn) assays, such as the European Society of Cardiology (ESC) 0/1-h algorithm and the High-STEACS pathway, given their excellent diagnostic performance.^2^^,^^3^

Different guidelines, however, recommend different diagnostic algorithms. The 2020 ESC guidelines for Non-ST-segment elevation acute coronary syndrome recommend the ESC 0/1-h and 0/2-h algorithms.^4^ However, they do not mention alternative algorithms or the reason for selecting these two. The 2021 American Heart Association/American College of Cardiology (ACC) guidelines for chest pain list the algorithms using history and physical findings and the hs-cTn-based but do not mention algorithm selection.^5^ The 2022 ACC Expert Consensus recommends hs-cTn-based algorithms such as the ESC 0/1-h, 0/2-h and High-STEACS pathways than algorithms using history and physical findings.^6^ However, which hs-cTn-based algorithm to use remains unclear. One reason is that limited prospective studies have validated and compared their performance in identical populations with uniform outcome definitions.^1^

The objective of this prospective multicenter cohort study was to validate the diagnostic algorithms for the early diagnosis of myocardial infarction using a high-sensitivity troponin I (hs-cTnI) assay in Japanese emergency departments where myocardial infarction due to coronary vasospasm is common.^7^^,^^8^ This study aimed to provide a comprehensive picture of early diagnostic algorithms for myocardial infarction and to aid clinicians in selecting a diagnostic algorithm.

## Materials and methods

### Study design and setting

We prospectively recruited patients with suspected myocardial infarction across nine emergency departments in Japan between July 2018 and July 2021. The hospital details are shown in Supplementary Table S1. We published the details of this study protocol previously.^9^

### Selection of participants

We included patients with symptoms suggestive of myocardial infarction, such as chest pain, within 6 hours of onset, who had no ST-segment elevation on the 12-lead electrocardiogram (ECG) at presentation. We excluded patients deemed eligible for emergency catheterization at presentation by a cardiologist, or whose time of onset was unknown. The details of the eligibility criteria are shown in Supplementary Data. This study was registered (UMIN 000029992), approved by the Ethics Committees of the Kyoto University Graduate School and Faculty of Medicine (R1380) and conducted following the Declaration of Helsinki. All patients provided written informed consent.

### Clinical assessments

We obtained baseline patient characteristics using standardized case record forms at the index visit. Emergency physicians recorded these findings before confirming the initial diagnosis. Serial cardiac troponin measurement on presentation and after 3 hours was at the physician’s discretion in standard care. Details of the standard care troponin assays used at each site are provided in Supplementary Table S1. We mandated to obtain research blood samples on presentation and 1, 2, and 3 hours after the first blood draw. The Abbott ARCHITECT STAT high-sensitive troponin I (Abbott Laboratories, Abbott Park, IL) assay was measured from the research blood samples to evaluate the diagnostic algorithms. Results of the research hs-cTnI assay were masked from the emergency physicians. Management of patients was at the physician’s discretion based on the results of standard care troponin assays, and structured algorithms were not used at the institutional level in any facility during the study. We reviewed medical records and conducted a structured telephone interview to obtain clinical outcomes within 30 days.

### High-sensitivity cardiac troponin I assay for research blood samples

We prepared serum samples for storage and measurement using the Abbott ARCHITECT STAT high-sensitive troponin I assay. The serum samples stored below −20°C at each hospital were sent to Abbott’s laboratory (Matsudo, Chiba, Japan) and measured by research staff blinded to the diagnosis. The assay has an inter-assay coefficient of variation of less than 10% at 4.7 ng/l, a uniform 99th percentile upper reference limit of 26.2 ng/l and a limit of detection of 1.2 ng/l.^10^

### Diagnostic algorithms

We assessed diagnostic algorithms with R (version 4.0.3) from prospectively collected clinical information. Hs-cTnI-based algorithms included the ESC 0/1-h, ESC 0/2-h, and High-STEACS pathways. The High-STEACS pathway is a 0 hours pathway optimized to rule out myocardial infarction at presentation but allows a second measurement at 2 hours if needed. Algorithms using medical history and physical findings included the ADAPT (2-Hour Accelerated Diagnostic Protocol to Assess Patients With Chest Pain Symptoms using Troponins), EDACS (Emergency Department Assessment of Chest Pain Score), HEART, and GRACE (Global Registry of Acute Coronary Events) pathways. The cut-off values for each algorithm were those listed in the guidelines.^4–6^ The details and thresholds for each algorithm are described in the Supplementary Material and protocol (Supplementary Tables S2–S5 and Figures S1–S3).^9^

### Clinical outcomes

The primary clinical outcome was the composite of type 1 myocardial infarction or cardiac death within 30 days. The diagnosis and classification of myocardial infarction were made following the fourth universal definition of myocardial infarction.^11^ Type 1 myocardial infarction was defined as myocardial infarction with suggestive symptoms of myocardial ischemia or results from cardiac investigations which confirmed myocardial ischemia. Type 2 myocardial infarction was defined as a condition other than coronary artery disease contributing to an oxygen supply-demand imbalance. The diagnosis was determined using coronary angiography or stress testing, or if not performed, coronary artery assessments before the index visit. Following the guidelines, vasospastic angina was defined when the patient had either transient ischemic ECG changes; or coronary artery spasm by provocation testing; or was responsive to nitrates and had the following features: onset during the night or early morning, attacks suppressed by calcium channel blockers but not beta blockers. When vasospastic angina was associated with myocardial injury, we diagnosed myocardial infarction due to coronary vasospasm.^12^ We defined cardiac death as any death due to myocardial infarction, such as heart failure or arrhythmia. Two cardiologists from each facility independently adjudicated the outcome based on all clinical and follow-up data using the results of research hs-cTnI assays taken at 0 and 3 hours or later as part of standard care. All cardiologists were masked from the results of the diagnostic algorithms. Agreement for the diagnosis of myocardial infarction was good (*κ* = 0.62; 95% confidence interval [CI], 0.52–0.73).

### Statistical analysis

We reported baseline patient characteristics as median (interquartile range [IQR]). We reported sensitivity and negative predictive value (NPV) as safety measures and positive predictive value (PPV) and proportion of patients classified as low or high-risk for efficiency measures. The Bayesian approach is robust in analyzing extreme values, and we used it with Jeffreys prior for estimating intervals because we expected the NPV to be close to 100%.^13^ This study was exploratory, and we reported 95% CI, not *P*-values.^14^ We used a clinical impression-based algorithm for sample size estimation, which encompasses risk assessment based on medical history and physical findings, the initial ECG, and troponin assays collected at 0 hours. Assuming that 5–10% of patients had the primary clinical outcome, with a sensitivity and specificity of 95% and 55%, respectively, 1500 patients were necessary to achieve the lower limit of 95% CI for the NPV was to surpass 98%.^9^^,^^15–17^ However, we stopped enrollment on July 2021 due to slow recruitment. We initially planned for consecutive cases, but in practice, it turned out to be a convenient sampling. We analyzed complete cases owing to the few missing values of baseline patient characteristics. Patients whose research blood samples were unavailable or not followed up for 30-day outcomes were excluded from the analysis. For the sensitivity analysis, we added type 2 myocardial infarction or myocardial infarction due to coronary vasospasm to the clinical outcome. We evaluated the diagnostic algorithms limited to patients whose research hs-cTnI assay was available at 0, 1 and 2 hours *post hoc*. All analyses were conducted with R (version 4.0.3).

## Results

### Characteristics of study subjects

**Figure 1.**
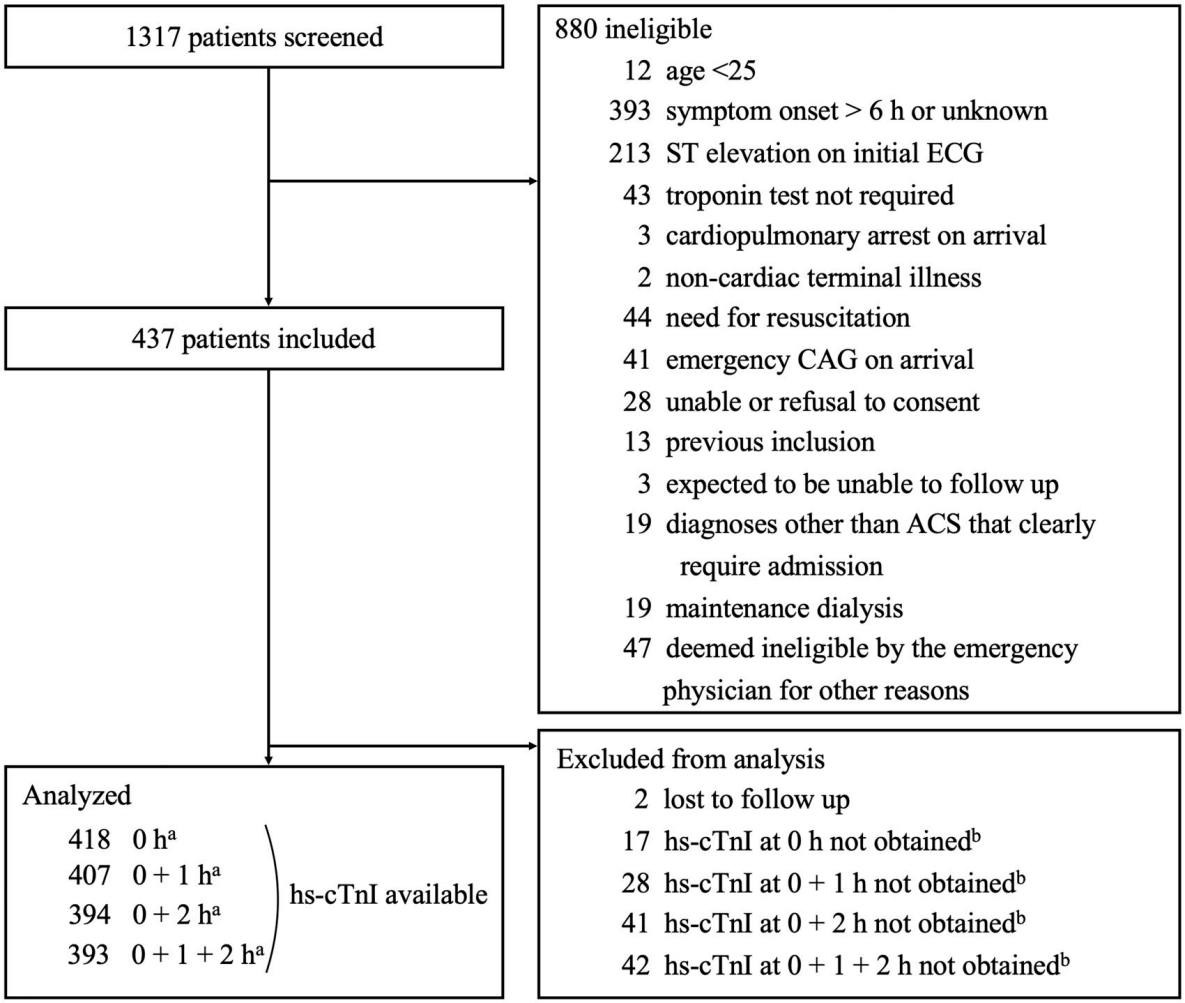
Patient flows. The research blood samples at presentation could not be collected from 17 patients due to overlooking to collect blood or mishandling. We stopped collecting research blood samples if patients had an emergency catheterization (deemed unnecessary at presentation but deemed necessary based on the course of events or troponin results afterwards) or hospitalization. ACS, acute coronary syndrome; CAG, coronary angiography; ECG, electrocardiography; hs-cTnI, high-sensitivity cardiac troponin I; h, hours. The sum of ^a^, ^b^ and 2 is 437.

We screened 1317 patients and excluded 880 patients for the following reasons: 393 had onset >6 hours, and 213 had ST-segment elevation on initial ECG (Figure 1). We included a total of 437 patients, with 2 were excluded due to an inability to follow up at 30 days. Of the remaining patients, the research hs-cTnI was collected at 0 and 1 hours in 407 patients (1 h group) and at 0 and 2 hours in 394 patients (2 h group). We used the 1-h group for the ESC 0/1-h algorithm and the 2-h group for all other algorithms. Patient characteristics and risk factors were similar in the 1- and 2-h groups: the median age was 72 years, 44.0% were female, and the median time from symptom onset to arrival was 1.5 hours (IQR, 1.0–3.0) (Table 1).

**Table 1.** Baseline characteristics

	1 h group (*n* = 407)	2 h group (*n* = 394)
Baseline characteristics		
Age, years	72.0 (58.0–82.0)	72.0 (57.3–81.8)
Female	179 (44.0)	175 (44.4)
Chest pain	357 (87.7)	345 (87.6)
Time from symptom onset, h	1.5 (1.0–3.0)	1.5 (1.0–3.0)
Risk factors		
Hypertension	284 (69.8)	275 (69.8)
Hyperlipidemia	215 (52.8)	209 (53.0)
Diabetes mellitus	110 (27.0)	105 (26.6)
Family history of premature CAD^a^	77 (19.0)	73 (18.6)
Current smoking^a^	73 (18.0)	71 (18.1)
Previous MI^a^	41 (10.1)	38 (9.7)
Previous VSA	43 (10.6)	43 (10.9)
Previous PCI	78 (19.2)	75 (19.0)
Medical history risk		
Highly suspicious	116 (28.5)	113 (28.7)
Moderately suspicious	215 (52.8)	207 (52.5)
Slightly suspicious	76 (18.7)	74 (18.8)
ECG findings		
New ST depression	25 (6.1)	23 (5.8)
New T-wave inversion	22 (5.4)	20 (5.1)
Physiological parameters		
Systolic BP, mmHg	143 (126–162)	143 (127–163)
Diastolic BP, mmHg	83 (75–94)	84 (75–94)
Heart rate, bpm	75 (67–86)	75 (67–85)
Creatinine clearance^b^^,^^c^	63.2 (43.5–93.4)	63.1 (43.5–93.5)
Hs-cTnI concentration at presentation, ng/l		
Type 1 MI	62.0 (16.0–432.0)	62.0 (16.0–432.0)
Type 2 MI	32.0 (13.8–47.8)	32.0 (13.8–47.8)
MI due to coronary vasospasm	17.0 (8.0–40.0)	17.0 (8.0–40.0)
no MI	4.0 (2.0–8.5)	4.0 (2.0–8.5)
Risk scores		
ADAPT	1 (1–3)	1 (1–3)
EDACS	19 (14–22)	19 (14–22)
GRACE	91 (63–108)	95 (69–115)
HEART	4 (3–5)	4 (3–5)

Values are median (interquartile range) or *n* (%).

a Data missing in one patient.

b ml/min/1.73 m^2^.

c Data missing in two patients.

BP, blood pressure; CABG, coronary artery bypass grafting; CAD, coronary artery disease; MI, myocardial infarction; PCI, percutaneous coronary intervention; VSA, vasospastic angina.

### Clinical outcomes

In the 1- and 2-h groups, respectively, 8.1% (33/407) and 6.9% (27/394) of patients had a primary outcome; 8.1% (33/407) and 6.9% (27/394) with type 1 myocardial infarction; 5.7% (23/407) and 5.3% (21/394) with type 2 infarction; and 3.2% (13/407) and 3.0% (12/394) myocardial infarction due to coronary vasospasm (Table 2). In 10 patients with the primary outcome, the initial hs-cTnI concentration was below the 99th percentile. Details of the cases are shown in Supplementary Table S6. The specifics of myocardial infarction due to coronary vasospasm cases are summarized in Supplementary Table S7.

**Table 2.** Managements and outcomes at 30 days

	1 h group (*n* = 407)	2 h group (*n* = 394)
Managements		
Stress ECG	20 (4.9)	19 (4.8)
Coronary CT	57 (14.0)	56 (14.2)
Coronary angiogram	101 (24.8)	92 (23.4)
PCI	55 (13.5)	39 (9.9)
CABG	1 (0.2)	1 (0.3)
Outcomes at 30 days		
Type 1 MI	33 (8.1)	27 (6.9)
Type 2 MI^a^	23 (5.7)	21 (5.3)
Type 2 MI due to coronary vasospasm	13 (3.2)	12 (3.0)
Ventricular arrhythmias requiring intervention^b^	3 (0.7)	2 (0.5)
Cardiac death^b^	1 (0.2)	1 (0.3)
Type 1 MI or cardiac death^c^	33 (8.1)	27 (6.9)
MI due to type 1 or coronary vasospasm or cardiac death^c^	46 (11.3)	39 (9.9)

a Coronary vasospasm included.

b Had type 1 MI at the index visit.

c Only the first event counted.

CABG, coronary artery bypass grafting; MI, myocardial infarction; PCI, percutaneous coronary intervention.

### Diagnostic performance of Hs-cTnI-based algorithms

#### ESC 0/1-h algorithm

**Figure 2.**
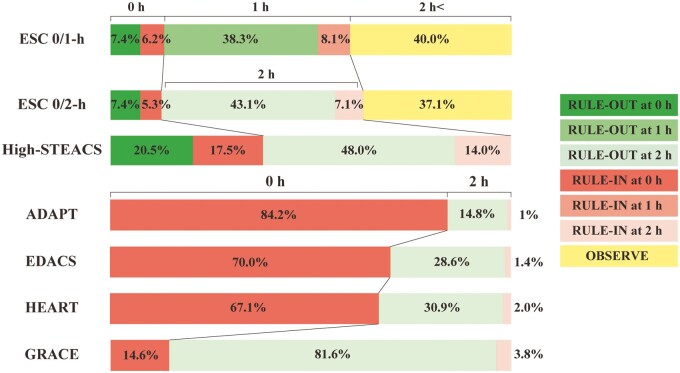
Classified timings and proportions. Timing and proportion of patients for rule-out, rule-in and not classified. ADAPT, 2-Hour Accelerated Diagnostic Protocol to Assess Patients With Chest Pain Symptoms using Troponins; EDACS, Emergency Department Assessment of Chest Pain Score; GRACE, Global Registry of Acute Coronary Events; HEART, History, Electrocardiography, Age, Risk factors, Troponin.

The ESC 0/1-h algorithm ruled out 45.7% (186/407) of the patients without missing any with type 1 myocardial infarction. The NPV was 99.7% (95% CI, 99.0–100.0), sensitivity 98.5% (95% CI, 94.4–100.0) and PPV 44.9% (95% CI, 32.5–57.6). The initial assessment classified 13.5% (55/407) of the patients, and the second assessment at 1 hour classified 46.5% (189/407). The remaining 40.0% (163/407) were not classified as either rule-out or rule-in (Table 3, Figure 2).

**Table 3.** Diagnostic performance for type 1 myocardial infarction or cardiac death

	True positive	False positive	True negative	False negative	Sensitivity (95% CI)	NPV (95% CI)	Rule out *n* (%)^a^	PPV (95% CI)	Rule inn (%)^b^	Observe *n* (%)^c^
ESC 0/1-h	26	32	186	0	98.5 (94.4–100.0)	99.7 (99.0–100.0)	186 (45.7)	44.9 (32.5–57.6)	58 (14.3)	163 (40.0)
ESC 0/2-h	25	24	199	0	98.2 (93.2–100.0)	99.8 (99.0–100.0)	199 (50.5)	51.0 (37.3–64.6)	49 (12.4)	146 (37.1)
High-STEACS	27	97	270	0	98.2 (93.2–100.0)	99.8 (99.3–100.0)	270 (68.5)	22.0 (15.2–29.6)	124 (31.5)	0
ADAPT	27	306	58	0	98.2 (93.2–100.0)	99.2 (96.8–100.0)	58 (14.8)	8.2 (5.5–11.4)	333 (85.2)	0
EDACS	27	252	112	0	98.2 (93.2–100.0)	99.6 (98.3–100.0)	112 (28.6)	9.8 (6.6–13.5)	279 (71.4)	0
HEART (0 and 2 h)	27	243	121	0	98.2 (93.2–100.0)	99.6 (98.4–100.0)	121 (30.9)	10.1 (6.8–14.0)	270 (69.1)	0
GRACE (0 and 2 h)	24	48	316	3	87.5 (73.2–96.8)	98.9 (97.5–99.7)	319 (81.6)	33.6 (23.3–44.7)	72 (18.4)	0

a Proportion of patients ruled out.

b Proportion of patients ruled in.

c Proportion of patients not classified as either rule-in or rule-out.

CI, confidence interval; NPV, negative predictive value; PPV, positive predictive value.

#### ESC 0/2-h algorithm

The ESC 0/2-h algorithm ruled out 50.5% (199/394) of the patients without missing any with type 1 myocardial infarction. The NPV was 99.8% (95% CI, 99.0–100.0), sensitivity 98.2% (95% CI, 93.2–100.0), and PPV 51.0% (95% CI, 37.3–64.6). The initial assessment classified 12.7% (50/394) of the patients, and the second assessment at 2 hours classified 50.2% (198/394). The remaining 37.1% (146/394) were not classified as rule-out or rule-in (Table 3, Figure 2).

#### High-STEACS pathway

The NPV was 99.8% (95% CI, 99.3–100.0), sensitivity 98.2% (95% CI, 93.2–100.0), and PPV 22.0% (95% CI, 15.2–29.6). Compared to the ESC 0/1-h and 0/2-h algorithms, the High-STEACS pathway ruled out more patients, 68.5% (270/394), without missing any with type 1 myocardial infarction (proportion difference 22.8% [95% CI, 16.1–29.4], 18.0% [95% CI, 11.2–24.7], respectively). Additionally, the initial assessment classified more patients than the ESC 0/1-h and 0/2-h algorithms, 38.1% (150/394), with a proportion difference of 24.6% (95% CI, 18.7–30.3) and 25.4% (95% CI, 19.5–31.1), respectively. No patients were classified to observe (Table 3, Figure 2).

### Diagnostic performance of algorithms using medical history and physical findings

#### ADAPT, EDACS, HEART pathways

The three pathways detected all the primary clinical events with NPVs and sensitivities above 99% and 98%, respectively, although all patients required assessment at 2 hours to rule out. The pathways ruled out a smaller proportion of patients, 14.8–30.9%, than those of the hs-cTnI-based algorithms (Table 3, Figure 2).

#### GRACE pathway

The GRACE pathway ruled out the largest patients, 81.6% (319/391), but missed three with type 1 myocardial infarction. The NPV was 98.9% (95% CI, 97.5–99.7), and the sensitivity was 87.5% (95% CI, 73.2–96.8) (Table 3, Figure 2). Details of the missed cases are shown in Supplementary Table S6.

#### Sensitivity analysis

When we added type 2 myocardial infarction or myocardial infarction due to coronary vasospasm to the primary outcome, we obtained similar results for the NPVs and sensitivities. The PPVs tended to be higher than for the primary outcome (Supplementary Tables S8 and S9). When we limited patients to those, the research hs-cTnI assay was available at all timings and applied all the algorithms to the identical population, the results were comparable (Supplementary Tables S10 and S11 and Figure S4).

## Limitations

Although we originally planned continuous sampling, this turned out to be convenient sampling in practice due to the slow recruitment and the coronavirus pandemic. However, the incidence of type 1 myocardial infarction or cardiac death within 30 days was comparable to the previous studies (4–20%).^16^ Furthermore, in more than half of the patients, the medical history risk was moderately suspicious, more so than in previous studies (30–39%).^18^^,^^19^ It was because very low-risk patients, for whom physicians would hesitate to follow up for 3 hours, and very high-risk patients, for whom cardiologists would be consulted immediately, were less likely to be included. Consequently, cases that were difficult to judge were more likely to be included, which provided a greater challenge for the performance of diagnostic algorithms. Despite this limitation, performance was good across most algorithms. Secondly, patient recruitment was slow, and the planned sample size could not be achieved, resulting in wide CIs for measures of sensitivity. However, the CIs for the NPV were narrow and clinically acceptable, enabling reliable conclusions based on this measure. Thirdly, we only included patients presented within 6 hours of onset, which may limit the generalizability of our results. However, in previous studies, early presenters consistently had lower or at least equal NPV and sensitivity than late presenters, making our results on safety more conservative.^2^^,^^3^^,^^16^^,^^20^^,^^21^ Lastly, this was an observational study, and the impact of these diagnostic algorithms on patient care and cost-effectiveness in this setting is unknown.

## Discussion

This was the first multicenter prospective cohort study in Japan to comprehensively validate the early diagnostic algorithms for myocardial infarction using an hs-cTnI assay. The hs-cTnI-based algorithms had excellent safety with consistently high NPV and sensitivities and were more efficient in identifying substantially more patients as low-risk than those using history and physical findings.

The ESC 0/1-h and 0/2-h algorithms classified 45.7% and 50.5% of patients as low-risk; 14.3% and 12.4% as high-risk, with the highest PPVs (44% and 51%), respectively. However, 40% and 37% of patients required further observation. The High-STEACS pathway classified the largest proportion of patients (69%) as low-risk and the remaining 31% as high-risk. It classified all patients as either ruled out or in at 2 hours. Whereas the PPVs were lower at 22% than those of the ESC 0/1-h and 0/2-h algorithms.

The main benefit of early diagnostic pathways is their potential to reduce time in the emergency department and avoid unnecessary hospital admissions. The High-STEACS pathway is primarily a single test rule-out pathway and, therefore, particularly effective, with 20.5% of patients in our study eligible for discharge following a single test, compared to 7.4% using the ESC algorithms. In the High-STEACS pathway, only those presenting early or with intermediate values between 5 ng/l and the 99th percentile require a second test in the emergency department; in our cohort, 62% of patients. However, most patients required a second test with the ESC 0/1-h and 0/2-h algorithms (86.4% and 87.3%, respectively). Although not directly comparable, the randomized trials evaluating the implementation of the High-STEACS pathway demonstrated a reduction in length of stay by 3.3 hours with 71% discharged from the emergency department, whereas the ESC 0/1-h algorithm reduced length of stay by 1 hour with 45% discharged.^22^^,^^23^

Consistent with the previous studies from Japan, our cohort was predominantly older patients and had a much short time between symptom onset and presentation. The incidence of type 1 and type 2 myocardial infarction was also similar to the studies (10.4, 5.6%, respectively), and there were more myocardial infarction due to coronary vasospasm compared to studies from Europe, USA, and Oceania.^16^^,^^24^^,^^25^

The NPV and the sensitivity of all the structured diagnostic algorithms were consistent with the original studies. In contrast, the proportions of low and high-risk patients differed.^2^^,^^26–30^ The ESC 0/1-h and 0/2-h algorithms in the original study classified 20% of patients as high-risk and 30% to observe, whereas in the present study, fewer patients were high-risk (12–14%), and more patients were classified to observe (40%). This may be because the cutoff concentration for the ESC 0/1-h rule-in algorithm (64 ng/l) was higher than in the original study (52 ng/l).^2^^,^^4^ The PPVs of the hs-TnI-based algorithms were lower than those of the original studies, likely reflecting the higher prevalence of type 2 myocardial infarction in our study. Indeed, in our sensitivity analysis for an outcome of type 1 or 2 myocardial infarction, the PPVs were similar to the original studies (Supplementary Table S8).^2^^,^^31^

To the best of our knowledge, this study is the first to evaluate the performance of structured diagnostic algorithms for all types of myocardial infarction, including those due to coronary vasospasm, which is more common in the Japanese population. However, further investigation in larger studies enrolling patients with coronary vasospasm is necessary, given the relatively small number of cases.

In summary, we prospectively and comprehensively evaluated the performance of popular diagnostic algorithms for the early diagnosis of myocardial infarction using an hs-cTnI assay. Among the diagnostic algorithms, those based on hs-cTnI measurements showed excellent performance for the early diagnosis of myocardial infarction and offer the greatest improvement in efficiency for clinical practice. Given that all the hs-cTnI algorithms worked well, as our study found, studies comparing the cost-effectiveness between these algorithms are warranted.^32^

## Supplementary Material

hcad242_Supplementary_Data
