# Supplementary material for: Comprehensive validation of early diagnostic algorithms for myocardial infarction in the emergency department
Source: QJM. 2023 Oct 25;117(3):187–94. doi: 10.1093/qjmed/hcad242 (PMC10972704; doi:10.1093/qjmed/hcad242)
Supplement: hcad242_Supplementary_Data [file hcad242_supplementary_data.docx]

**Supplemental material**

**Comprehensive validation of very early diagnostic algorithms for myocardial infarction in the emergency department**

Authors: Masafumi Tada, MD; Hideyuki Matano, MD; Hiroyuki Azuma, MD; Ken-ichi Kano, MD; Shigenobu Maeda, MD; Susumu Fujino, MD, PhD; Naoki Yamada, MD; Hiroyasu Uzui, MD, PhD; Hiroshi Tada, MD, PhD; Koji Maeno, MD, PhD; Yoshimitsu Shimada, MD; Hiroyuki Yoshida, MD, PhD; Masaki Ando, MD; Taku Ichihashi, MD, PhD; Yoshimasa Murakami, MD, PhD; Yosuke Homma, MD, MPH; Hiraku Funakoshi, MD, MPH, PhD; Kotaro Obunai, MD, PhD; Asako Matsushima, MD, PhD; Nobuyuki Ohte, MD, PhD; Akinori Takeuchi, MD, PhD; Yasunobu Takada, MD, PhD; Shohei Matsukubo, MD, MBA; Hirotaka Ando, MD; Yoshio Furukawa, MD, PhD; Akira Kuriyama, MD, PhD; Takeshi Fujisawa, MD, PhD; Andrew R. Chapman, MD, PhD; Nicholas L. Mills, MD, PhD; Hiroyuki Hayashi, MD; Norio Watanabe, MD, PhD; Toshi A Furukawa, MD, PhD.

[Supplemental Data. Eligibility Criteria 3](#_Toc148589998)

[Supplemental Table 1. Troponin assays used at each site 4](#_Toc148589999)

[Supplemental Table 2. ADAPT pathway 5](#_Toc148590000)

[Supplemental Table 3. EDACS pathway 6](#_Toc148590001)

[Supplemental Table 4. HEART pathway 7](#_Toc148590002)

[Supplemental Table 5. GRACE pathway 8](#_Toc148590003)

[Supplemental Table 6. Summary of patients with primary outcome and initial hs-cTnI concentration below the 99^th^ percentile 9](#_Toc148590004)

[Supplemental Table 7. Patients with myocardial infarction due to coronary vasospasm 10](#_Toc148590005)

[Supplemental Table 8. Diagnostic performance for type 1 or 2 myocardial infarction or cardiac death within 30 days 12](#_Toc148590006)

[Supplemental Table 9. Diagnostic performance for type 1 or myocardial infarction due to coronary vasospasm or cardiac death within 30 days 13](#_Toc148590007)

[Supplemental Table 10. Baseline characteristics, managements and outcomes at 30 days for patients with research hs-cTnI collected at 0, 1 and 2 hours 14](#_Toc148590008)

[Supplemental Table 11. Diagnostic performance for type 1 myocardial infarction or cardiac death within 30 days in patients the research hs-cTnI assay was available at 0, 1 and 2 hours 16](#_Toc148590009)

[Supplemental Figure 1. ESC 0/1-h algorithm 17](#_Toc148590010)

[Supplemental Figure 2. ESC 0/2-h algorithm 18](#_Toc148590011)

[Supplemental Figure 3. High-STEACS pathway 19](#_Toc148590012)

[Supplemental Figure 4. Classified timings and proportions in patients the research hs-cTnI assay was available at 0, 1 and 2 hours 20](#_Toc148590013)

# **Supplemental** **Data. Eligibility Criteria**

When an emergency physician suspected myocardial infarction during an examination, the physician immediately evaluated the patient's eligibility for the study according to the following criterion.

**Inclusion criteria**

1. Age ≥25 years
2. Have any symptoms suggestive of myocardial infarction
3. Presentation to the emergency department within 6 hours from symptom onset
4. No apparent ST elevation on arrival
5. An emergency physician deemed both an ECG and a troponin assay necessary

**Exclusion criteria**

1. Cardiopulmonary arrest on arrival.
2. Non-cardiac terminal illness (expected survival < 6 months)
3. Need for resuscitation
4. Indication of emergency catheterisation on arrival
5. Patients unable to provide consent
6. Previous inclusion in the study
7. Patients who are expected to be difficult to follow up after 30 days due to dementia, psychiatric disorders, living alone, or not having a phone
8. Unclear onset time of symptoms
9. The Apparent need to admit for a diagnosis other than acute coronary syndrome on arrival
10. Patients on maintenance dialysis
11. An emergency physician judges ineligible due to other reasons, such as dementia or psychiatric disorders that make it challenging to obtain a medical history, uncooperative in medical examinations, or other diseases, such as neurally mediated syncope or gallstones, are strongly suspected, and myocardial infarction is very unlikely

| **Supplemental Table 1. Troponin assays used at each site** | |
| --- | --- |
| **Hospital** | **Troponin assay** |
| Nagoya City University Hospital | Sysmex HISCL Troponin T h |
| Fukui Prefectural Hospital^a^ | Radiometer AQT90 FLEX Troponin T |
| Fukuiken Saiseikai Hospital^b^ | Radiometer AQT90 FLEX Troponin T, Abbott ARCHITECT hs-cTnI^c^ |
| Fukui University Hospital | Radiometer AQT90 FLEX Troponin T, Abbott ARCHITECT hs-cTnI^d^ |
| Ichinomiyanishi Hospital^b^ | Abbott ARCHITECT hs-cTnI |
| Japanese Red Cross Fukui Hospital^b^ | Abbott ARCHITECT hs-cTnI |
| Konan Kosei Hospital^a^ | Abbott ARCHITECT hs-cTnI |
| Nagoya City East Medical Center^a^ | Abbott ARCHITECT hs-cTnI |
| Tokyo Bay Urayasu Ichikawa Medical Center^a^ | Abbott ARCHITECT hs-cTnI |
| ^a^Tertiary level, ^b^Secondary level  ^c^Changed from the troponin T to the hs-cTnI assay in June 2020 | |
| ^d^Changed from the troponin T to the hs-cTnI assay in June 2019 | |

| **Supplemental Table 2. ADAPT pathway** | | |
| --- | --- | --- |
|  | 1. Hs-cTnI level at 0 and 2 hours below the 99^th^ percentile value | |
|  | 2. No new ischemic changes on the initial ECG | |
|  | 3. TIMI score = 0^a^ | |
|  |  | a. Age ≧65 years |
|  |  | b. Three or more risk factors for coronary artery disease:   (family history of coronary artery disease, hypertension, hypercholesterolaemia,   diabetes, current smoker) |
|  |  | c. Use of aspirin in the past seven days |
|  |  | d. Significant coronary stenosis (e.g. previous coronary stenosis >50%) |
|  |  | e. Severe angina (e.g. ≧2 angina events in past 24 h or persisting discomfort) |
|  |  | f. ST-segment deviation of ≧0.05 mV on first ECG |
|  |  | g. Increased hs-cTnI level at 0 h |
| ^a^All items, a to g, do not apply | | |
| All parameters have to be negative to be considered at low risk | | |

| **Supplemental Table 3. EDACS pathway** | | | | |  |
| --- | --- | --- | --- | --- | --- |
|  | 1. No red flags: abnormal vital signs, pain that is ongoing or in a crescendo pattern | | | |  |
|  | 2. No new ischemic changes on the initial ECG | | | |  |
|  | 3. Hs-cTnI level at 0 and 2 hours below the 99^th^ percentile value | | | |  |
|  | 4. EDACS score <16 | | | |  |
|  |  | a. Age range, y | | Points | |
|  |  |  | 18 - 45 | | 2 |
|  |  |  | 46 - 50 | | 4 |
|  |  |  | 51 - 55 | | 6 |
|  |  |  | 56 - 60 | | 8 |
|  |  |  | 61 - 65 | | 10 |
|  |  |  | 66 - 70 | | 12 |
|  |  |  | 71 - 75 | | 14 |
|  |  |  | 76 - 80 | | 16 |
|  |  |  | 81 - 85 | | 18 |
|  |  |  | >86 | | 20 |
|  |  | b. Only if age 18-50y | | |  |
|  |  |  | Known CAD or ≧3 of the following 5 CAD risk factors: family history of CAD, dyslipidemia, diabetes, hypertension, current smoker | | 4 |
|  |  | c. Symptoms | | |  |
|  |  |  | Diaphoresis (in association with pain) | | 3 |
|  |  |  | Pain radiates to arm or shoulder | | 5 |
|  |  |  | Pain occurs or worsened with inspiration (pleuritic in nature) | | -4 |
|  |  |  | Pain reproduced by palpation | | -6 |
|  |  | d. Sex | | |  |
|  |  |  | Male | | 6 |
| CAD = coronary artery disease | | | | |  |
| All parameters have to be negative to be considered at low risk | | | | |  |

| **Supplemental Table 4. HEART pathway** | |  | |
| --- | --- | --- | --- |
|  |  | | Points |
| History | Highly suspicious | | 2 |
|  | Moderately suspicious | | 1 |
|  | Slightly suspicious | | 0 |
|  |  | |  |
| ECG | Significant ST depression | | 2 |
|  | Nonspecific repolarization disturbance | | 1 |
|  | Normal | | 0 |
|  |  | |  |
| Age, y | ≥65 | | 2 |
|  | 45 - 64 | | 1 |
|  | <45 | | 0 |
|  |  | |  |
| Risk factors | ≥3 risk factors or history of atherosclerotic disease | | 2 |
|  | 1 or 2 risk factors | | 1 |
|  | No risk factors known | | 0 |
|  |  | |  |
| Troponin | >2× 99^th^ percentile | | 2 |
|  | 1-2× 99^th^ percentile | | 1 |
|  | < 99^th^ percentile | | 0 |

HEART score <4 and hs-cTnI level at 0 and 2 hours below the 99^th^ percentile value is considered at low risk

| **Supplemental Table 5. GRACE pathway** | | | | | | | | | | | | | | | | | | |
| --- | --- | --- | --- | --- | --- | --- | --- | --- | --- | --- | --- | --- | --- | --- | --- | --- | --- | --- |
| Medical history | | | |  | |  | Findings at initial hospital presentation | | | | | Findings during hospitalization | | | | | | |
|  | 1. Age, y | | |  | | Points |  | 4. Resting heart rate | | | Points |  | 7. Initial serum creatinine | | | | | Points |
|  |  | | ≤29 |  | | 0 |  |  | ≤49.9 | | 0 |  |  | 0 - 0.39 | | |  | 1 |
|  |  | | 30 - 39 |  | | 0 |  |  | 50 - 69.9 | | 3 |  |  | 0.4 - 0.79 | | |  | 3 |
|  |  | | 40 - 49 |  | | 18 |  |  | 70 - 89.9 | | 9 |  |  | 0.8 - 1.19 | | |  | 5 |
|  |  | | 50 - 59 |  | | 36 |  |  | 90 - 109.9 | | 14 |  |  | 1.2 - 1.59 | | |  | 7 |
|  |  | | 60 - 69 |  | | 55 |  |  | 110 - 149.9 | | 23 |  |  | 1.6 - 1.99 | | |  | 9 |
|  |  | | 70 - 79 |  | | 73 |  |  | 150 - 199.9 | | 35 |  |  | 2 - 3.99 | | |  | 15 |
|  |  | | 80 - 89 |  | | 91 |  |  | ≥200 | | 43 |  |  | ≥4 | | |  | 20 |
|  |  | | ≥90 |  | | 100 |  | 5. Systolic blood pressure, mm Hg | | | |  | 8. Elevated cardiac enzymes | | | | | 15 |
|  | 2. History of congestive heart failure | | | | | 24 |  |  | ≤79.9 | | 24 |  | 9. No in-hospital PCI | | | | | 14 |
|  | 3. History of myocardial infarction | | | | | 12 |  |  | 80 - 99.9 | | 22 |  |  | |  | |  |  |
|  |  |  | |  | |  |  |  | 100 - 119.9 | | 18 |  |  | |  | |  |  |
|  |  |  | |  | |  |  |  | 120 - 139.9 | | 14 |  |  | |  | |  |  |
|  |  |  | |  | |  |  |  | 140 - 159.9 | | 10 |  |  | |  | |  |  |
|  |  |  | |  | |  |  |  | 160 - 199.9 | | 4 |  |  | |  | |  |  |
|  |  |  | |  | |  |  |  | ≥200 | | 0 |  |  | |  | |  |  |
|  |  |  | |  | |  |  | 6. ST-segment depression | | | 11 |  |  | |  |  | |  |
| PCI = percutaneous intervention | | | | |  | |  |  |  |  |  |  |  | |  | |  |  |

GRACE score <140 and hs-cTnI levels at 0 and 2 hours below the 99^th^ percentile is considered at low risk

| **Supplemental Table 6. Summary of patients with primary outcome and initial hs-cTnI concentration below the 99^th^ percentile** | | | | | | | | | | | | | | |
| --- | --- | --- | --- | --- | --- | --- | --- | --- | --- | --- | --- | --- | --- | --- |
| **Age** | **Gender** | **Symptom** | **Time from  onset (h)** | **Hs-cTnI (ng/L)** | | | | **Diagnosis** | **New ischemic ECG findings** | **TIMI**  **Score** | **EDACS**  **Score** | **GRACE**  **Score** | **HEART Score** | **Comments** |
|  |  |  |  | **0 h** | **1 h** | **2 h** | **3 h** |  |  |  |  |  |  |  |
| 77 | F | Chest pain | 2.0 | 10 | 56 | 373 | 925 | Index Type 1 MI | No | 2 | 19 | 95 | 6 |  |
| 63 | M | Chest pain | 1.3 | 1 | 84 | 281 | 545 | Index Type 1 MI | No | 0 | 24 | 79 | 3 |  |
| 77 | F | Chest pain | 4.0 | 23 | 55 | 103 | 264 | Index Type 1 MI | No | 2 | 19 | 79 | 5 |  |
| 73 | F | Chest pain | 0.7 | 4 | 5 | 109 | 137 | Index Type 1 MI | No | 2 | 17 | 95 | 3 |  |
| 78 | M | Chest pain | 1.5 | 14 | 30 | 65 | 192 | Index Type 1 MI | No | 2 | 25 | 97 | 7 |  |
| *72 | M | Chest pain | 0.5 | 16 | 14 | 15 | 17 | Type 1 MI (30d) | Yes | 2 | 23 | 93 | 5 | Admitted due to ischemic ECG changes. Hs-cTnI got positive the next morning and underwent PCI |
| 67 | M | Nausea, syncope | 2.4 | 5 | 9 | 54 | 179 | Index Type 1 MI | Yes | 4 | 21 | 80 | 6 |  |
| *71 | M | Chest pain | 1.0 | 5 | 6 | 9 | 2 | Type 1 MI (30d) | No | 2 | 20 | 89 | 3 | Discharged that day, but chest pain recurred the next day and was admitted to the hospital with NSTEMI and underwent PCI |
| *70 | M | Chest pain | 1.0 | 3 | 9 | 26 | 55 | Index Type 1 MI | No | 1 | 21 | 102 | 4 |  |
| 83 | M | Chest pain | 1.0 | 15 | 56 | 156 | 314 | Index Type 1 MI | No | 2 | 32 | 109 | 5 |  |

Index Type 1 MI = Type 1 myocardial infarction diagnosed at the index visit

Type 1 MI (30d) = Type 1 myocardial infarction diagnosed within 30 days (not at the index visit)

*Missed cases using the GRACE pathway

| **Supplemental Table 7. Patients with myocardial infarction due to coronary vasospasm** | | | | | | | | | | | | | |
| --- | --- | --- | --- | --- | --- | --- | --- | --- | --- | --- | --- | --- | --- |
| **Age** | **Gender** | **Symptom** | **Time from  onset (h)** | **Hs-cTnI (ng/L)** | | | | **Diagnosis of VSA** | **Risk  factors** | **Initial ECG** | **Provocation** | **CAG** | **Coronary CT** |
|  |  |  |  | **0 h** | **1 h** | **2 h** | **3 h** |  |  |  |  |  |  |
| 53 | M | chest pain | 1.0 | 17.3 | 57.0 | 66.9 | 156.1 | definite | HTN, HL, DM,  smoker | SR | Ach + | No stenosis | No stenosis |
| 70 | M | chest pain | 1.4 | 8.1 | 28.3 | 103.4 | 260.1 | definite | HTN, HL, DM,  ex-smoker, Previous MI and PCI | New lateral  ST depression | Ach + | No stenosis | Not  performed |
| 45 | M | chest pain | 2.3 | 7.9 | 14.1 | 32.8 | NA | definite | HTN, HL, DM, smoker,  Previous MI and PCI, VSA | SR | Ach + | #3, 75%; #4, 75% (no change) | Not  performed |
| 48 | M | chest pain | 0.8 | 3.7 | 32.9 | 113.8 | 183.7 | definite | HTN, HL,  ex-smoker, VSA | SR | Ach + | No stenosis | Not  performed |
| 69 | M | chest pain | 3.0 | 51.1 | 53.5 | 51.2 | NA | definite | HTN, HL, smoker,  Previous MI and PCI, VSA | SR | Ach + | #D1, 75% (no change) | Not  performed |
| 54 | M | chest pain | 2.5 | 53.1 | 72.7 | 93.3 | NA | definite | HTN, HL, DM,  ex-smoker, Previous MI due to definite VSA | SR | Not  performed | No stenosis (performed 1 year ago) | Not  performed |
| 38 | M | chest pain | 1.0 | 2.3 | 9.5 | 33.2 | 58.0 | suspected | HTN, smoker | SR | Not  performed | Not  performed | No stenosis |

Ach = acetylcholine; CAG = coronary angiography; CT = computed tomography; DM = diabetes mellitus; h = hours; HL = hyperlipidemia; HTN = hypertension; MI = myocardial infarction; NA = not available; PCI = percutaneous coronary intervention; SR = sinus rhythm; VSA = vasospastic angina

**Supplemental Table 7. Continued**

| 54 | M | chest pain | 2.0 | 203.3 | 276.5 | 427.7 | NA | definite | HTN, smoker | New anterolateral  T-wave inversion | Not  performed | No stenosis | Not  performed |
| --- | --- | --- | --- | --- | --- | --- | --- | --- | --- | --- | --- | --- | --- |
| 74 | M | chest pain | 1.0 | 12.6 | 82.6 | 152.7 | NA | definite | ex-smoker | New diffuse  T-wave inversion | Not  performed | #6, 75%; #12, 75% (FFR negative) | Not  performed |
| 61 | M | chest pain | 0.2 | 31.1 | 33.1 | NA | NA | definite | HTN, HL, | SR | Ergonovine + | No stenosis | Not performed |
| 72 | M | chest pain | 2.0 | 16.2 | 29.5 | 47.2 | 63.9 | suspected | HTN, HL,  ex-smoker,  VSA | SR | Not performed | Not  performed | Not  performed |
| 67 | M | chest pain | 4.0 | 40.0 | 64.1 | 67.8 | 87.6 | suspected | HTN, smoker | Known anterolateral T-wave inversion | Not performed | Not  performed | No  stenosis |
| 49 | F | chest pain | 2.0 | 26.0 | 126.2 | 313.1 | 613.3 | suspected | None | SR | Not performed | Not  performed | No  stenosis |

Ach = acetylcholine; CAG = coronary angiography; CT = computed tomography; DM = diabetes mellitus; FFR = fractional flow reserve; h = hours; HL = hyperlipidemia; HTN = hypertension; MI = myocardial infarction; NA = not available; PCI = percutaneous coronary intervention; SR = sinus rhythm; VSA = vasospastic angina

| **Supplemental Table 8. Diagnostic performance for type 1 or 2 myocardial infarction or cardiac death within 30 days** | | | | | | | | | |  |
| --- | --- | --- | --- | --- | --- | --- | --- | --- | --- | --- |
|  | **True  Positive** | **False  Positive** | **True  Negative** | **False  Negative** | **Sensitivity (95% CI)** | **NPV (95% CI)** | **Rule Out  (%)**^a^ | **PPV (95% CI)** | **Rule In  (%)**^b^ | **Observe n (%)**^c^ |
| **ESC 0/1-h** | 45 | 13 | 186 | 0 | 99.1  (96.6-100.0) | 99.7  (99.0-100.0) | 186 (45.7) | 77.1  (65.7-86.8) | 58 (14.3) | 163 (40.0) |
| **ESC 0/2-h** | 43 | 6 | 199 | 0 | 99.0  (96.1-100.0) | 99.8  (99.0-100.0) | 199 (50.5) | 87.0  (76.5-94.7) | 49 (12.4) | 146 (37.1) |
| **High-STEACS** | 47 | 77 | 269 | 1 | 96.9  (90.7-99.8) | 99.4  (98.3-100.0) | 270 (68.5) | 38.0  (29.7-46.6) | 124 (31.5) | 0 |
| **ADAPT** | 48 | 285 | 58 | 0 | 99.0  (96.1-100.0) | 99.2  (96.8-100.0) | 58 (14.8) | 14.5  (11.0-18.5) | 334 (85.2) | 0 |
| **EDACS** | 48 | 231 | 112 | 0 | 99.0  (96.1-100.0) | 99.6  (98.3-100.0) | 112 (28.6) | 17.3  (13.1-22.0) | 280 (71.4) | 0 |
| **HEART (0 and 2 h)** | 47 | 223 | 120 | 1 | 96.9  (90.7-99.8) | 98.8  (96.2-99.9) | 121 (30.9) | 17.5  (13.2-22.3) | 271 (69.1) | 0 |
| **GRACE (0 and 2 h)** | 43 | 29 | 314 | 5 | 88.8  (78.7-95.9) | 98.3  (96.6-99.4) | 319 (81.6) | 59.6  (48.2-70.5) | 72 (18.4) | 0 |

CI = confidence interval; NPV = negative predictive value; PPV = positive predictive value.

^a^Proportion of patients ruled out. ^b^Proportion of patients ruled in. ^c^Proportion of patients not classified as either rule-in or rule-out.

| **Supplemental Table 9. Diagnostic performance for type 1 or myocardial infarction due to coronary vasospasm or cardiac death within 30 days** | | | | | | | | | |  |
| --- | --- | --- | --- | --- | --- | --- | --- | --- | --- | --- |
|  | **True  Positive** | **False  Positive** | **True  Negative** | **False  Negative** | **Sensitivity (95% CI)** | **NPV (95% CI)** | **Rule Out  (%)**^a^ | **PPV (95% CI)** | **Rule In  (%)**^b^ | **Observe n (%)**^c^ |
| **ESC 0/1-h** | 37 | 21 | 186 | 0 | 98.9  (95.9-100.0) | 99.7  (99.0-100.0) | 186 (45.7) | 63.6  (51.0-75.3) | 58 (14.3) | 163 (40.0) |
| **ESC 0/2-h** | 36 | 13 | 199 | 0 | 98.8  (95.2-100.0) | 99.8  (99.0-100.0) | 199 (50.5) | 73.0  (60.0-84.2) | 49 (12.4) | 146 (37.1) |
| **High-STEACS** | 39 | 85 | 270 | 0 | 98.8  (95.2-100.0) | 99.8  (99.3-100.0) | 270 (68.5) | 31.6  (23.8-40.0) | 124 (31.5) | 0 |
| **ADAPT** | 39 | 294 | 58 | 0 | 98.8  (95.2-100.0) | 99.2  (96.8-100.0) | 58 (14.8) | 11.8  (8.6-15.5) | 334 (85.2) | 0 |
| **EDACS** | 39 | 240 | 112 | 0 | 98.8  (95.2-100.0) | 99.6  (98.3-100.0) | 112 (28.6) | 14.1  (10.3-18.4) | 280 (71.4) | 0 |
| **HEART (0 and 2 h)** | 39 | 231 | 121 | 0 | 98.8  (95.2-100.0) | 99.6  (98.4-100.0) | 121 (30.9) | 14.6  (10.6-19.0) | 271 (69.1) | 0 |
| **GRACE (0 and 2 h)** | 36 | 36 | 316 | 3 | 91.3  (80.9-97.8) | 98.9  (97.5-99.7) | 319 (81.6) | 50.0  (38.6-61.4) | 72 (18.4) | 0 |

CI = confidence interval; NPV = negative predictive value; PPV = positive predictive value.

^a^Proportion of patients ruled out. ^b^Proportion of patients ruled in. ^c^Proportion of patients not classified as either rule-in or rule-out.

# **Supplemental Table 10. Baseline characteristics, managements and outcomes at 30 days for patients with research hs-cTnI collected at 0, 1 and 2 hours**

|  | **0+1+2 h group**  **(n = 393)** | |
| --- | --- | --- |
| Baseline characteristics | | |
| Age, y | | 72.0 (57.0-81.0) |
| Female | | 174 (44.3) |
| Chest pain | | 344 (87.5) |
| Time from symptom onset, hours | | 1.5 (1.0-3.0) |
| Risk factors | | |
| Hypertension | | 274 (69.7) |
| Hyperlipidemia | | 208 (52.9) |
| Diabetes mellitus | | 105 (26.7) |
| Family history of premature CAD^a^ | | 73 (18.6) |
| Current smoking^a^ | | 71 (18.1) |
| Previous MI^a^ | | 38 (9.7) |
| Previous VSA | | 43 (10.9) |
| Previous PCI | | 75 (19.1) |
| Medical history risk | | |
| Highly suspicious | | 113 (28.8) |
| Moderately suspicious | | 206 (52.4) |
| Slightly suspicious | | 74 (18.8) |
| ECG findings | | |
| New ST depression | | 23 (5.9) |
| New T-wave inversion | | 20 (5.1) |
| Physiological parameters | |  |
| Systolic BP, mm Hg | | 143 (127-162) |
| Diastolic BP, mm Hg | | 83 (75-94) |
| Heart rate, bpm | | 75 (67-85) |
| Creatinine clearance^b,c^ | | 63.1 (43.4-93.5) |
| Hs-cTnI concentration at presentation^d^ | |  |
| Type 1 MI | | 62.0 (14.5.0-431.5) |
| Type 2 MI | | 32.0 (13.0-44.0) |
| MI due to coronary vasospasm | | 16.5 (8.0-42.8) |
| no MI | | 4.0 (2.0-9.0) |

**Supplemental Table 10. Continued**

| Risk scores | |  |
| --- | --- | --- |
| ADAPT | 1 (1-3) | |
| EDACS | 19 (14-22) | |
| GRACE | 95 (69-115) | |
| HEART | 4 (3-5) | |
| Managements | | |
| Coronary CT | 55 (14.0) | |
| Coronary angiogram | 92 (23.4) | |
| PCI | 39 (9.9) | |
| CABG | 1 (0.3) | |
| Outcomes at 30 days | | |
| Type 1 MI | 27 (6.9) | |
| Type 2 MI^e^ | 21 (5.3) | |
| Type 2 MI due to coronary vasospasm | 12 (3.1) | |
| Cardiac death | 1 (0.3) | |
| Type 1 MI or cardiac death^f^ | 27 (6.9) | |
| MI due to type 1 or coronary vasospasm or cardiac death^f^ | 39 (9.9) | |

Values are median (interquartile range) or n (%).

BP = blood pressure; CABG = coronary artery bypass grafting; CAD = coronary artery disease; MI = myocardial infarction; PCI = percutaneous coronary intervention; VSA = vasospastic angina.

^a^Data missing in 1 patient. ^b^ml/min/1.73 m^2^. ^c^Data missing in 2 patients. ^d^ng/L. ^e^Coronary vasospasm included. ^f^Only the first event counted.

| **Supplemental Table 11. Diagnostic performance for type 1 myocardial infarction or cardiac death within 30 days in patients the research hs-cTnI assay was available at 0, 1 and 2 hours** | | | | | | | | | |  |
| --- | --- | --- | --- | --- | --- | --- | --- | --- | --- | --- |
|  | **True  Positive** | **False  Positive** | **True  Negative** | **False  Negative** | **Sensitivity (95% CI)** | **NPV (95% CI)** | **Rule Out  (%)**^a^ | **PPV (95% CI)** | **Rule In  (%)^b^** | **Observe n (%)^c^** |
| **ESC 0/1-h** | 22 | 31 | 184 | 0 | 98.2  (93.2-100.0) | 99.7  (99.0-100.0) | 184 (46.8) | 41.7  (29.0-54.9) | 53 (13.5) | 156 (39.7) |
| **ESC 0/2-h** | 25 | 24 | 199 | 0 | 98.2  (93.2-100.0) | 99.8  (99.0-100.0) | 199 (50.6) | 51.0  (37.3-64.6) | 49 (12.5) | 145 (36.9) |
| **High-STEACS** | 27 | 97 | 269 | 0 | 98.2  (93.2-100.0) | 99.8  (99.3-100.0) | 269 (68.4) | 22.0  (15.2-29.6) | 124 (31.6) | 0 |
| **ADAPT** | 27 | 305 | 58 | 0 | 98.2  (93.2-100.0) | 99.2  (96.8-100.0) | 58 (14.9) | 8.3  (5.5-11.4) | 332 (85.1) | 0 |
| **EDACS** | 27 | 251 | 112 | 0 | 98.2  (93.2-100.0) | 99.6  (98.3-100.0) | 112 (28.7) | 9.9  (6.6-13.6) | 278 (71.3) | 0 |
| **HEART (0 and 2 h)** | 27 | 242 | 121 | 0 | 98.2  (93.2-100.0) | 99.6  (98.4-100.0) | 121 (31.0) | 10.2  (6.9-14.1) | 269 (69.0) | 0 |
| **GRACE (0 and 2 h)** | 24 | 48 | 315 | 3 | 87.5  (73.2-96.8) | 98.9  (97.5-99.7) | 318 (81.5) | 33.6  (23.3-44.7) | 72 (18.5) | 0 |

CI = confidence interval; NPV = negative predictive value; PPV = positive predictive value.

^a^Proportion of patients ruled out. ^b^Proportion of patients ruled in. ^c^Patients not classified as either rule-in or rule-out.

# **Supplemental Figure 1. ESC 0/1-h algorithm**

**
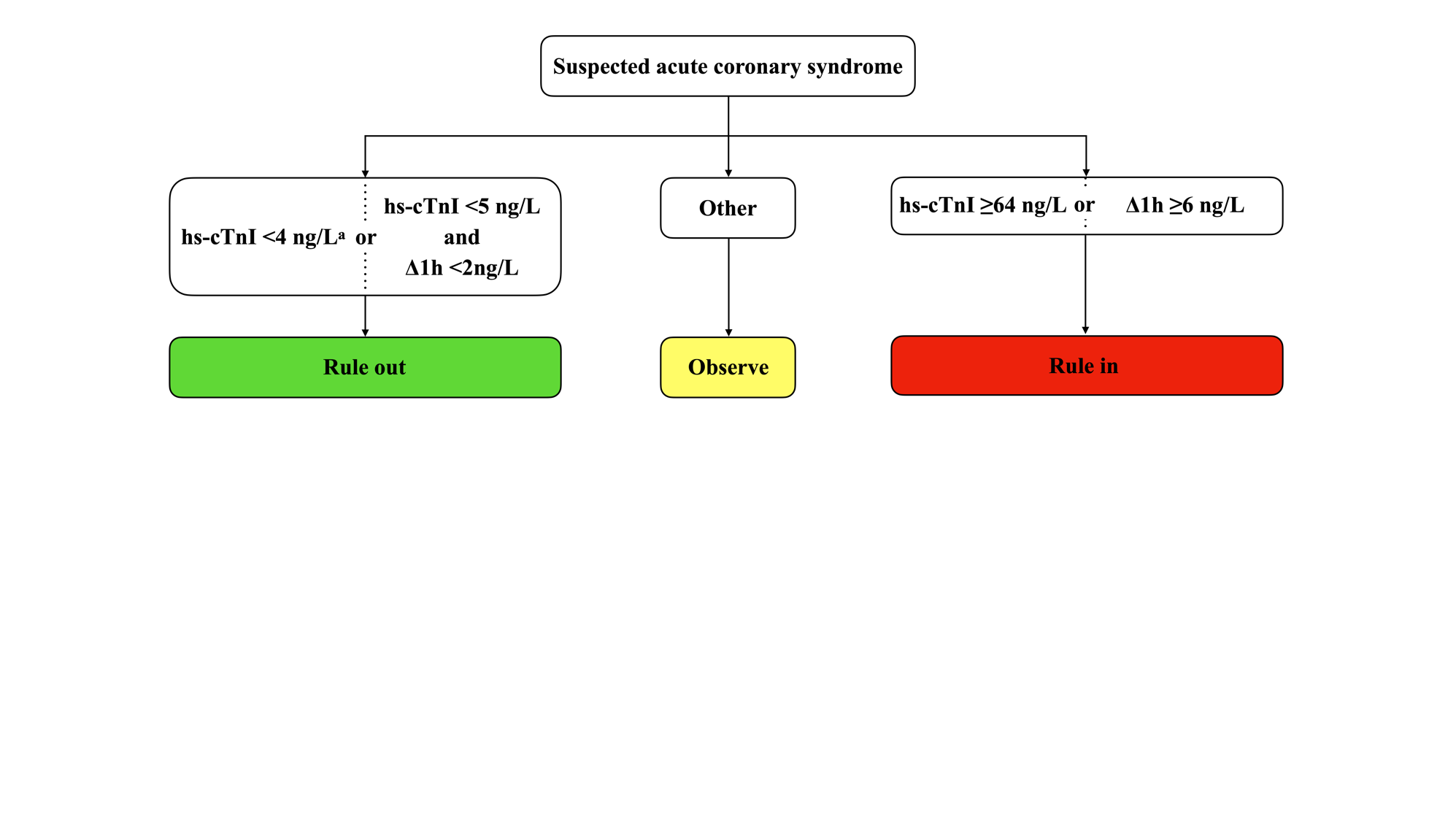
**

^a^Only applicable if symptom onset >3 hours

# **Supplemental Figure 2. ESC 0/2-h algorithm**

**
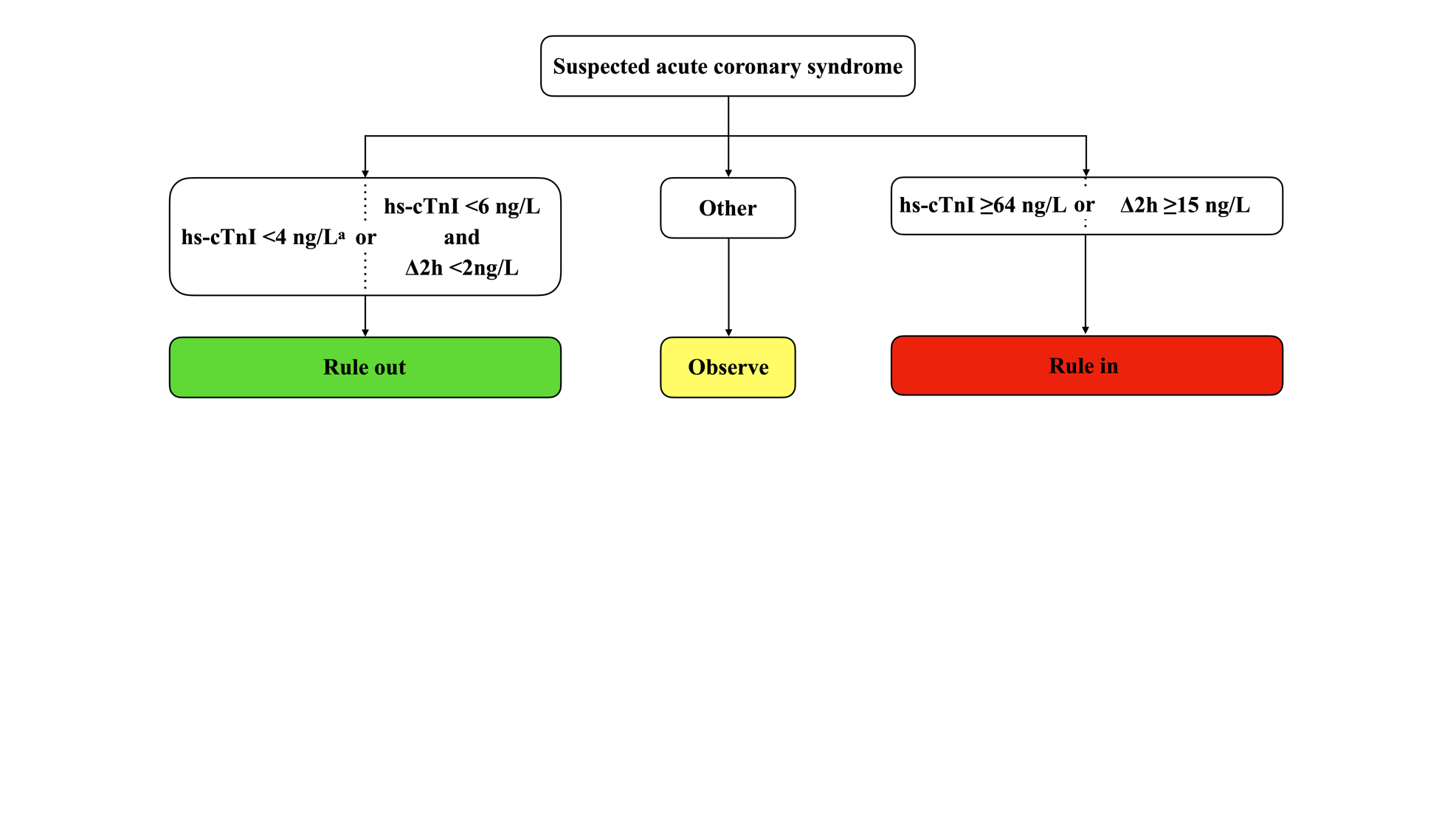
**

^a^Only applicable if symptom onset >3 hours

# **Supplemental Figure 3. High-STEACS pathway**


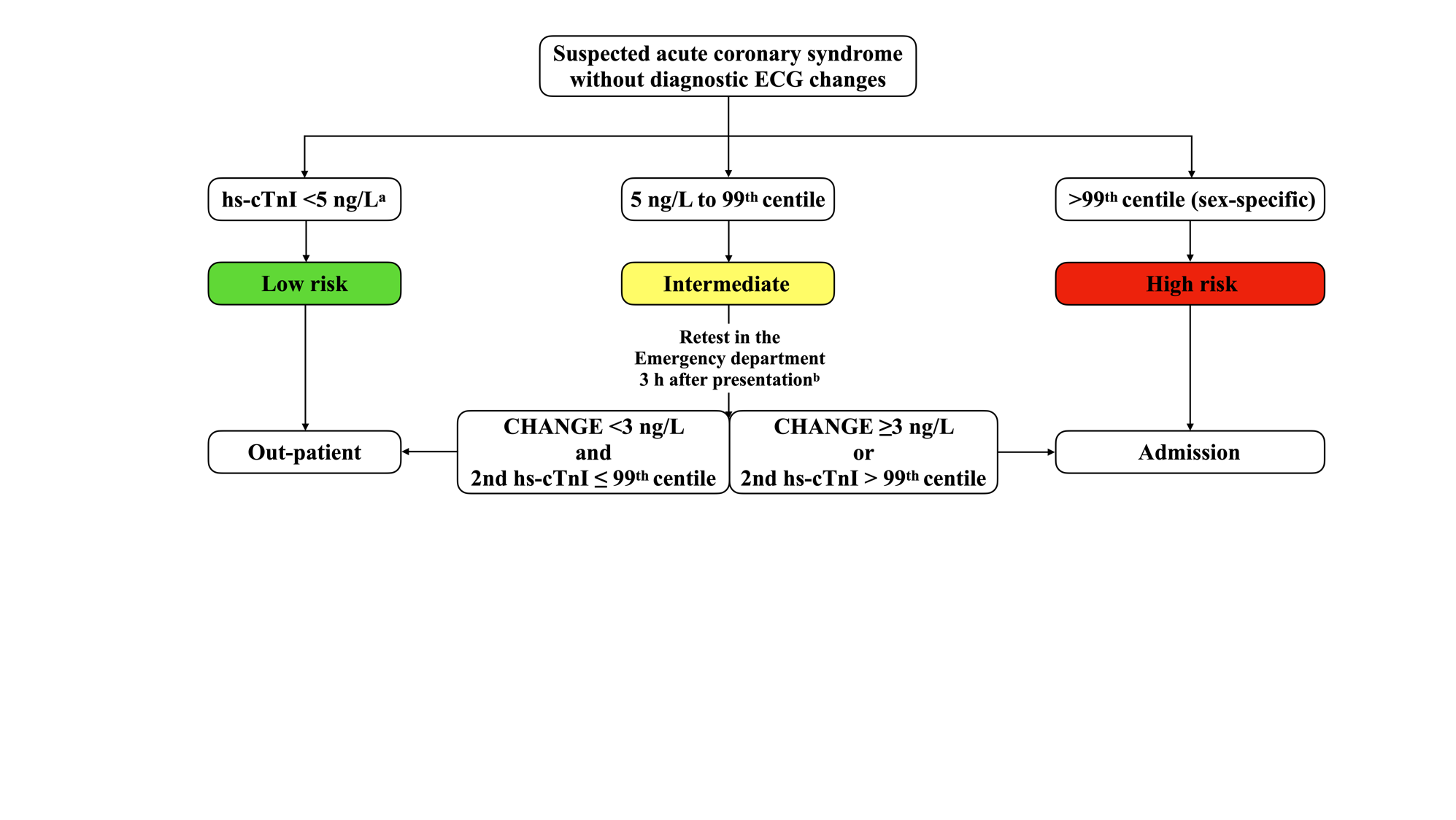


^a^Retest at 3 hours if ≤2 hours from symptom onset

^b^The original study took approximately 1 hour from the presentation to the 0 h blood draw. Therefore, a blood draw 3 hours after the presentation is equivalent to 2 hours after the 0 h blood draw

**Supplemental Figure 4. Classified timings and proportions in patients the research hs-cTnI assay was available at 0, 1 and 2 hours**


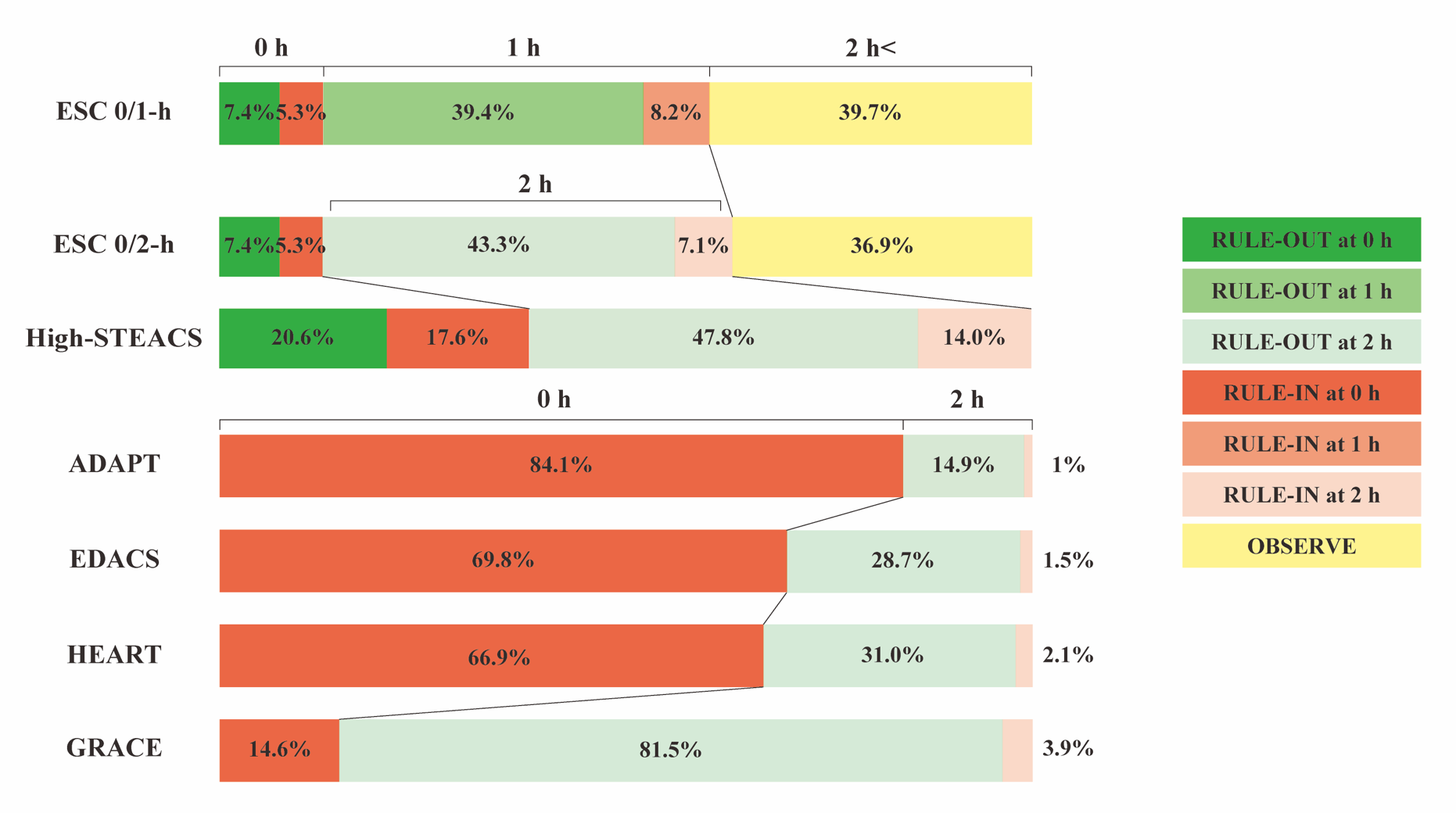


Timing and proportion of patients for rule-out, rule-in and not classified in patients with the research hs-cTnI assay was available at all timings (0, 1 and 2 hours).

ADAPT = 2-Hour Accelerated Diagnostic Protocol to Assess Patients With Chest Pain Symptoms using Troponins; EDACS = Emergency Department Assessment of Chest Pain Score; GRACE = Global Registry of Acute Coronary Events; HEART = History, Electrocardiography, Age, Risk factors, Troponin
